# Optimal mask fixation method for frameless radiosurgery with Leksell Gamma Knife Icon^TM^

**DOI:** 10.1002/acm2.13892

**Published:** 2023-01-07

**Authors:** Hyeong Cheol Moon, Hyun‐Tai Chung, Byung Jun Min, Yun‐Sik Dho

**Affiliations:** ^1^ Department of Neurosurgery Chungbuk National University Hospital Cheongju Republic of Korea; ^2^ Department of Neurosurgery Seoul National University Hospital Seoul Republic of Korea; ^3^ Department of Medical Device Development Seoul National University College of Medicine Seoul Republic of Korea; ^4^ Department of Radiation Oncology Chungbuk National University Hospital Cheongju Republic of Korea; ^5^ Department of Neurosurgery Chungbuk National University College of Medicine Cheongju Republic of Korea

**Keywords:** Gamma Knife radiosurgery, mask fixation methods, thermoplastic mask

## Abstract

The Leksell Gamma Knife (LGK) Icon^TM^ is used for mask‐based and frame‐based fixation. The mask fixation provides a noninvasive method. However, an optimal mask fixation method is yet to be established. We evaluated the characteristics of three mask fixation methods (Plain, Folded, and Wide) for the LGK Icon^TM^. Force‐sensitive resistor sensors were attached to the forehead, supraorbital, zygoma, mandible, and occipital bone of the phantom, and digital humidity and temperature sensors were attached to both temporal lobes. Cone‐beam computed tomography (CBCT) and high‐definition motion management (HDMM) for each mask fixation method were used to evaluate the phantom motion during the initial application. Subsequently, the mask was removed and reapplied on the second (1st reapplication) and third days (2nd reapplication). In the initial application, forces acting on most portions of the phantom were stabilized within 1.5 h. The largest force acted on the occipital bone for the Plain and Wide methods and on the mandible for the Folded method. The temperature rapidly approaches the initial temperature, whereas the humidity gradually approached the initial humidity in all fixation methods. The Folded method exhibited a significantly lower translation along the Y‐axis of the Leksell coordinate system, and rotations along all axes were under 0.5°. The HDMM values remained at 0.1 mm for all fixation methods. In the reapplications, the force acting on the occipital bone was significantly greater than that during the initial application for all mask fixation methods; the temperature and humidity remained unchanged. All mask fixation methods in the 1st reapplication were not significantly different from those in the 2nd reapplication. The Folded method is recommended as an optimal mask fixation for patients who require tight fixation; the Wide method can be considered if patient comfort is a priority.

## 1 INTRODUCTION

Gamma Knife radiosurgery (GKRS) has been used to treat brain tumors, vascular malformations, and other abnormalities in the brain. The Leksell Gamma Knife (LGK) Icon^TM^ was introduced in 2016 as an updated version of the existing the LGK Perfexion^TM^ model.^1^ In GKRS, a head frame is rigidly attached to the skull of a patient to establish a coordination system and enable precise positioning.^2^ Fixation of the frame on the skull is crucial in the LGK Perfexion^TM^. However, most patients experience considerable discomfort during this process.^2^ Common reasons for the denial of frame fixation by patients include anxiety, intolerance against local anesthetics, or lack of stability of the skull.^3^ With the advent of frameless radiosurgery options in the LGK Icon^TM^, a thermoplastic mask has been adopted for immobilization as it does not require an invasive frame. Therefore, the LGK Icon^TM^, which can facilitate frameless radiosurgery, has rendered fractionated GKRS an increasingly favorable treatment alternative.^4^

The LGK Icon^TM^ includes cone‐beam computed tomography (CBCT), and it can define a three‐dimensional (3D) stereotactic coordinate system for frame treatments or for a new frameless thermoplastic mask system. The accuracy of the thermoplastic mask is maintained within the submillimeter range by combining pretreatment CBCT imaging and co‐registration and via the monitoring of intra‐fractional motions using a high‐definition motion management (HDMM) system.^5^, ^6^ The HDMM system is coupled with a thermoplastic mask using an infrared camera and reflective nose marker.^7^, ^8^ However, the treatment time can be increased if the motion in the nose marker exceeds a physicist‐set threshold in the HDMM system (default of 1.5 mm but will allow any value up to 3 mm); this can lead to increased stress on the medical staff and patients. Several factors such as exceeding threshold in the HDMM system, patient's condition, mask discomfort, and overheated X‐ray tube in CBCT can cause an increase in treatment time. Compared with the Efficast mask (Orfit Industries, Wijnegem, Belgium), the recently developed Nanor mask (Orfit Industries, Wijnegem, Belgium) improves patient comfort by decreasing shrinkage and pressure. Patients have reported that the Nanor mask feels considerably tighter on the second day. The curing characteristics of thermoplastic fixation have been examined using a head mannequin model.^9^ Based on our experience, the Nanor mask shrinks over time, and the head cushion (Moldcare; Alcare Co, Tokyo, Japan) hardens; thus, the forces acting on the face and occipital bone appear to increase over time. This makes it difficult for patients to undergo prolonged GKRS, given the increasing force acting on the head owing to mask fixation. Therefore, identifying an optimal method for mask application is necessary to achieve firm fixation and ensure patient comfort.

**FIGURE 1:**
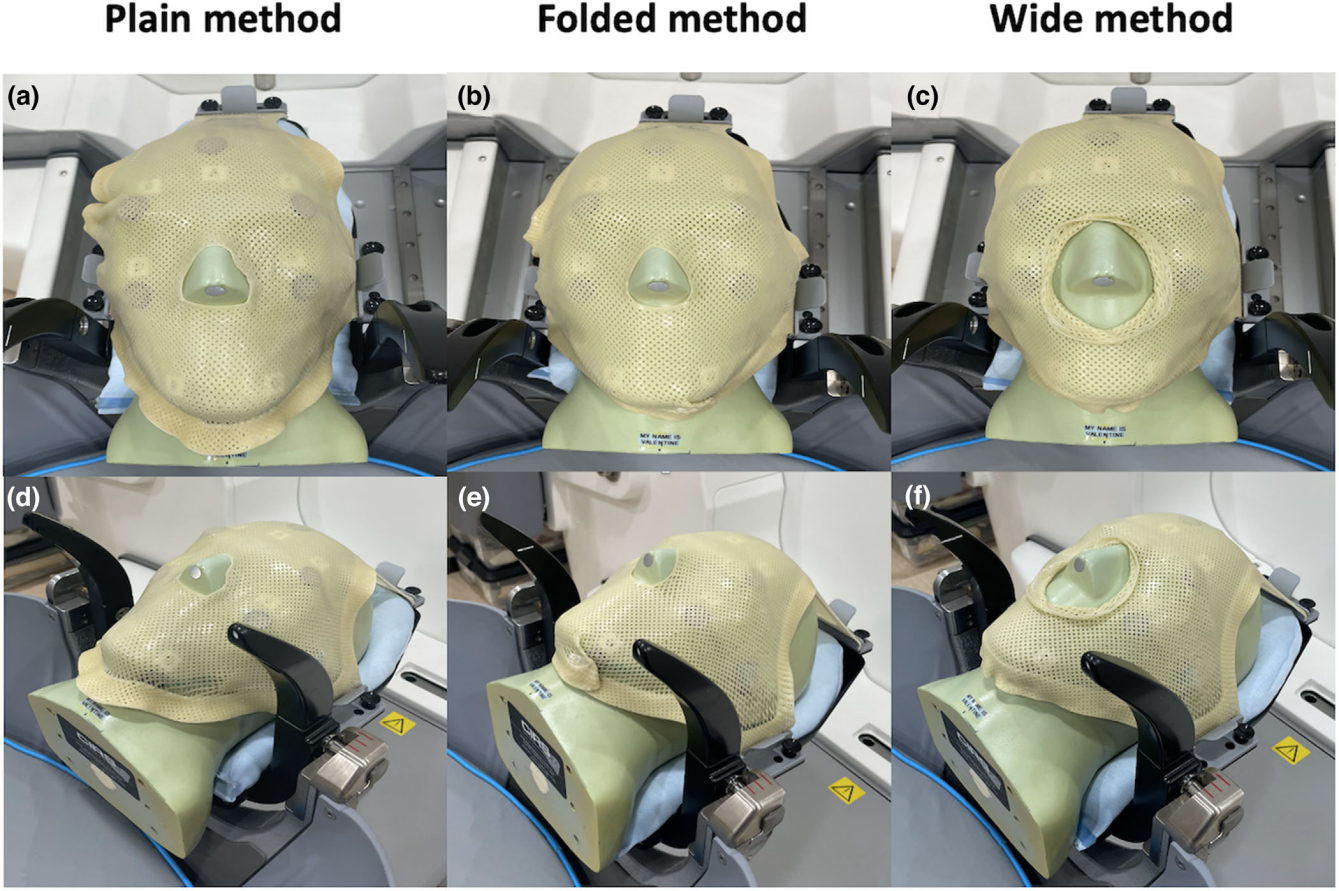
Three methods for mask fixation. Plain (a, d), Folded (b, e), and Wide (c, f) methods

In this study, we investigated the force, temperature, and humidity at various portions of an anthropomorphic phantom (CIRS Radiosurgery Head Phantom Model605, CIRSInc., Norfolk, VA, USA), on which a mask was fixed using three different mask fixation methods (Plain, Folded, and Wide; Figure 1). Further, we investigated inter‐ and intra‐fractional motion for suitable motion control of each mask fixation method. We evaluated the characteristics of the three kinds of mask fixation methods for 3 days (initial application, 1st reapplication, and 2nd reapplication) to determine an optimal mask fixation method for LGK Icon^TM^.

## 2 METHODS AND MATERIALS

### 2.1 Preparation

**FIGURE 2:**
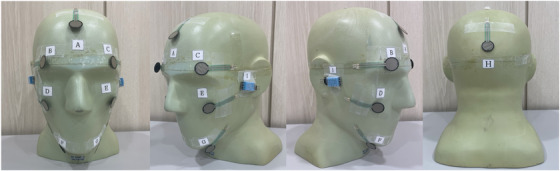
Head phantom with force‐sensitive sensors attached to the forehead (a), supraorbital (b, c), zygoma (d, e), mandible (f, g), and occipital bone (h). Digital humidity and temperature sensors attached to both temporal lobes (i, j)

We used a commercial anthropomorphic phantom to investigate the characteristics of each mask fixation method. A force‐sensing resistor (FSR) (402 FSR, Interlink Electronics, USA) sensor is employed in various industries as it is easy to use and reliable for pressure measurement.^10^ The FSR sensor is a thick‐film polymer device that exhibits a decrease in its resistance with an increase in the force applied onto its active surface.^11^ The output of the FSR sensor is related to the applied force, load resistance across the FSR (10 kOhms) sensor, and voltage applied to the measuring circuit (5 V); the force‐sensitive range is 0.1–100 N. For our analysis, we selected FSR sensor positions based on painful sites identified by patients during mask fixation, including the forehead, supraorbital, zygoma, mandible, and occipital bone (Figure 2). Furthermore, a digital humidity and temperature (DHT) (DHT 11, Electronics, USA) sensor was employed. Generally, high temperature and humidity can make it difficult for patients to breathe, which can lead to increased oxygen demand and feelings of tiredness and breathlessness. Conventionally, DHT sensors need to be attached near the nose; however, the mask could be deformed by the sensor, and therefore, it was attached to the temporal lobe (Figure 2).

Arduino is an open‐source platform used for simulating and programming electronics;^12^ it includes a circuit board with a programmable chip that can perform several tasks. Information can be sent from a computer program to the Arduino microcontroller and finally to a specific circuit or machine with multiple circuits to execute commands.^12^, ^13^ For our analysis, 10 Arduino Uno boards were connected to a MacBook Pro (Intel Core i9, 8 Cores, 16 GB RAM). Before mask fixation, serial data acquisition was initiated, and appropriate signal communication was verified. The average treatment time in the GKRS during mask fixation was set to approximately 1 h.^5^ Considering the preparation time, the time for real‐time data acquisition was set to 1.5 h. Serial data were continuously monitored and recorded using the CoolTerm software (version 1.9.0) for 1.5 h. The captured serial data were exported as Excel files; the data were extracted every 10 min and were exported to GraphPad Prism (GraphPad 9.2.0) for statistical analyses.

### 2.2 Mask fixation

A total of nine Nanor masks were fixed in three different methods (Plain, Folded, and Wide) (Figure 1). Three masks were fixed with each method. Generally, the Plain method is a baseline mask fixation method that does not result in mask deformation; the Folded method provides strong fixation, in which the mask is folded according to the shape of the mandible, and the Wide method provides loose fixation with the opening of the nose to ensure patient comfort. The phantom was placed on a head cushion, which was sprayed with water, and the shape was adjusted according to the occipital bone. Each mask was heated for 10 min in water at 60°C and then dried using a towel. The mask was placed on the phantom by two operators (medical physicist and nurse). The mask alone was carefully removed and placed in a Gamma Knife treatment room after data acquisition was completed; the phantom was left in the mask adapter. The mask was reapplied on the second (1st reapplication) and third days (2nd reapplication) after the first day (Initial application). A precision air conditioner controlled the temperature (20–23°C) and humidity (35%–40%) in the Gamma Knife treatment room.

### 2.3 Inter‐ and intra‐fraction motion

The motion of the phantom for each mask fixation method was examined using CBCT imaging and the HDMM system. Planning CBCT imaging (weighted CT dose index (CTDI_w_) = 6.3 mGy) was performed immediately after mask fixation, and pretreatment CBCT imaging (CTDI_w_ = 2.5 mGy) was performed every 30 min. A co‐registration system was used to evaluate the rotations and translations of the phantom between CBCT imagings.^14^ Co‐registration of the planning CBCT images was used to evaluate the inter‐fraction motions of each mask fixation method. Further, the translational intra‐fraction motion of the phantom was monitored in real‐time using the HDMM system. The HDMM value was recorded every 10 min.

### 2.4 Statistical analyses

The data were analyzed using GraphPad Prism. One‐way analysis of variance (ANOVA) was performed via Kruskal–Wallis and Dunn's post hoc tests for the fixation methods. We used a simple linear regression model to confirm the relationship between the acquired data and acquisition time. The results are presented as mean ± standard deviation; further, a difference is considered significant when the *p*‐value is less than 0.05.

## 3 RESULTS

### 3.1 Forces acting during initial application

**FIGURE 3:**
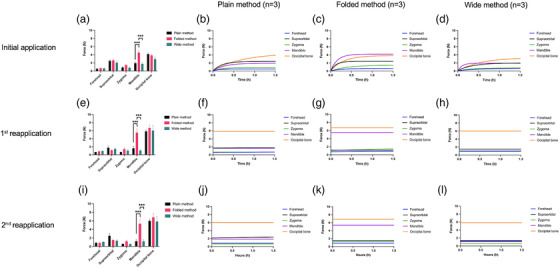
Forces acting on various portions of the phantom under the three different fixation methods. One‐way ANOVA (Dunn's post hoc test) is used to assess statistical differences (***p < 0.001)

In the initial application, forces acting on most portions of the phantom stabilized within 1.5 h, except those for the Plain and Wide methods (Figure 3). The fastest force stabilization was achieved for the Folded method (Figure 3c), where the forces acting on the supraorbital and mandible were stabilized within 30 min. The largest force was measured on the occipital bone for the Plain and Wide methods and on the mandible for the Folded method. The forces stabilized within 1.5 h; the force acting on the mandible for the Folded method (4.51 ± 1.19 N) was significantly greater than that for the Plain (1.93 ± 0.73 N, ***p < 0.001) and Wide methods (1.76 ± 0.87 N, ***p < 0.001). In the other portions, no significant difference was observed in the forces for each mask fixation method.

### 3.2 Forces acting during reapplications

The forces acting on all portions of the phantom stabilized immediately after mask reapplication (Figure 3). During the 1st reapplication, the force acting on the mandible for the Folded method (5.50 ± 2.34 N) was significantly greater than that for the Plain (1.61 ± 1.07 N, ***p < 0.001) and Wide methods (1.06 ± 0.54 N, ***p < 0.001). During the 2nd reapplication, the force acting on the mandible for the Folded method (5.33 ± 2.73 N) was significantly greater than that for the Plain (1.75 ± 0.95 N, ***p < 0.001) and Wide methods (1.22 ± 0.91 N, ***p < 0.001).

**FIGURE 4:**
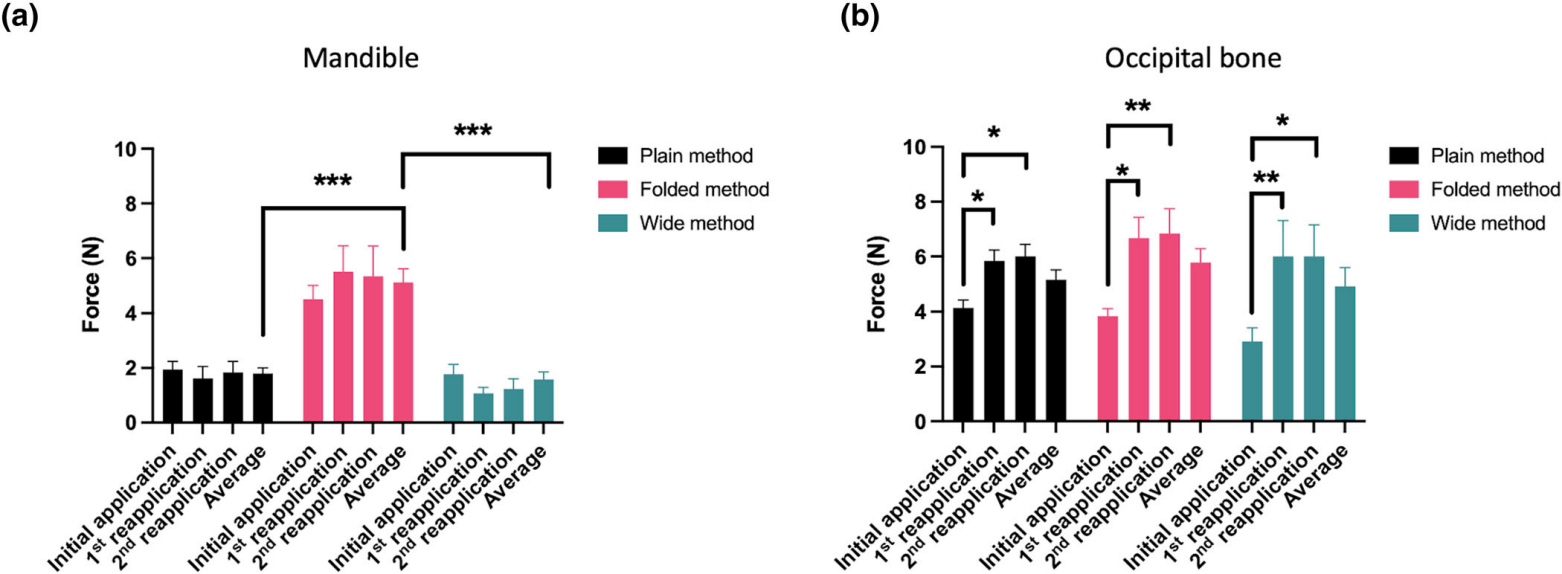
Comparison of the forces for each mask fixation method for 3 days. The average in each graph represents the average value of the force for 3 days. Forces acting on the mandible (a). Forces acting on the occipital bone (b). One‐way ANOVA (Dunn's post hoc test) is used to assess statistical differences (*p < 0.05, **p < 0.01, ***p < 0.001)

We compared the forces corresponding to each mask fixation method for 3 days. The average force acting on the mandible for 3 days for the Folded method (5.13 ± 2.21 N) was greater than that for the Plain (1.84 ± 0.89 N, ***p < 0.001) and Wide (1.41 ± 0.80 N, ***p < 0.001) methods (Figure 4a). The forces acting on the occipital bone for each mask fixation method revealed significant differences for 3 days (Table 1, Figure 4b). All mask fixation methods demonstrated large forces acting on the occipital bone in the reapplications compared to those in the initial application; the average force acting on the occipital bone for 3 days revealed no significant difference for any mask fixation method. Similarly, other portions did not exhibit significant differences in force changes for 3 days.

**TABLE 1:** Comparison of forces in the initial application, 1st reapplication, and 2nd reapplication for each mask fixation method

	Plain method (*n* = 3)						Folded method (*n* = 3)						Wide method (*n* = 3)					
	Initial vs. 1st Reapplication	*P*‐value	Initial vs. 2nd Reapplication	*P*‐value	1st Reapplication vs. 2nd reapplication	*P*‐value	Initial vs. 1st Reapplication	*P*‐value	Initial vs. 2nd Reapplication	*P*‐value	1st Reapplication vs. 2nd Reapplication	*P*‐value	Initial vs. 1st Reapplication	*P*‐value	Initial vs. 2nd Reapplication	*P*‐value	1st Reapplication vs. 2nd Reapplication	*P*‐value
Forehead	0.52 ± 0.25	NS	0.52 ± 0.25	NS	0.67 ± 0.01	NS	0.66 ± 0.28	NS	0.66 ± 0.28	NS	0.83 ± 0.28	NS	0.64 ± 0.27	NS	0.64 ± 0.27	NS	0.93 ± 0.11	NS
	0.67 ± 0.01		0.85 ± 0.21		0.85 ± 0.21		0.83 ± 0.28		0.80 ± 0.26		0.80 ± 0.26		0.93 ± 0.11		1.00 ± 0.01		1.00 ± 0.01	
Supraorbital	2.51 ± 0.52	NS	2.51 ± 0.52	NS	1.73 ± 1.01	NS	2.60 ±. 0.71	NS	2.60 ±. 0.71	NS	1.13 ± 0.42	NS	2.04 ± 1.08	NS	2.04 ± 1.08	NS	1.45 ± 0.60	NS
	1.73 ± 1.01		2.25 ± 1.50		2.25 ± 1.50		1.13 ± 0.42		1.50 ± 0.57		1.50 ± 0.57		1.45 ± 0.60		1.25 ± 0.50		1.25 ± 0.50	
Zygoma	0.87 ± 0.32	NS	0.87 ± 0.32	NS	0.67 ± 0.05	NS	1.46 ± 0.52	NS	1.46 ± 0.52	NS	1.41 ± 0.64	NS	0.82 ± 0.35	NS	0.82 ± 0.35	NS	1.01 ± 0.64	NS
	0.67 ± 0.05		0.63 ± 0.01		0.63 ± 0.01		1.41 ± 0.64		1.12 ± 0.58		1.12 ± 0.58		1.01 ± 0.64		0.61 ± 0.25		0.61 ± 0.25	
Mandible	1.93 ± 0.73	NS	1.93 ± 0.73	NS	1.61 ± 1.07	NS	4.51 ± 1.19	NS	4.51 ± 1.19	NS	5.50 ± 2.34	NS	1.76 ± 0.87	NS	1.76 ± 0.87	NS	1.06 ± 0.54	NS
	1.61 ± 1.07		1.75 ± 0.95		1.75 ± 0.95		5.50 ± 2.34		5.33 ± 2.73		5.33 ± 2.73		1.06 ± 0.54		1.22 ± 0.91		1.22 ± 0.91	
Occipital bone	4.13 ± 0.02	*0.03	4.13 ± 0.02	*0.02	5.83 ± 2.33	NS	3.83 ± 0.76	*0.04	3.83 ± 0.76	**0.005	6.66 ± 2.08	NS	2.84 ± 1.26	**0.008	2.84 ± 1.26	*0.02	6.00 ± 3.60	NS
	5.83 ± 2.33		6.00 ± 1.41		6.00 ± 1.41		6.66 ± 2.08		6.83 ± 2.82		6.83 ± 2.82		6.00 ± 3.60		5.86 ± 3.21		5.86 ± 3.21	

*Note*: *P*‐values calculated using Kruskal–Wallis and Dunn's post hoc tests.

Abbreviation: NS, not significant.

*p < 0.05, **p < 0.01.

### 3.3 Inter‐ and intra‐fraction motion

**FIGURE 5:**
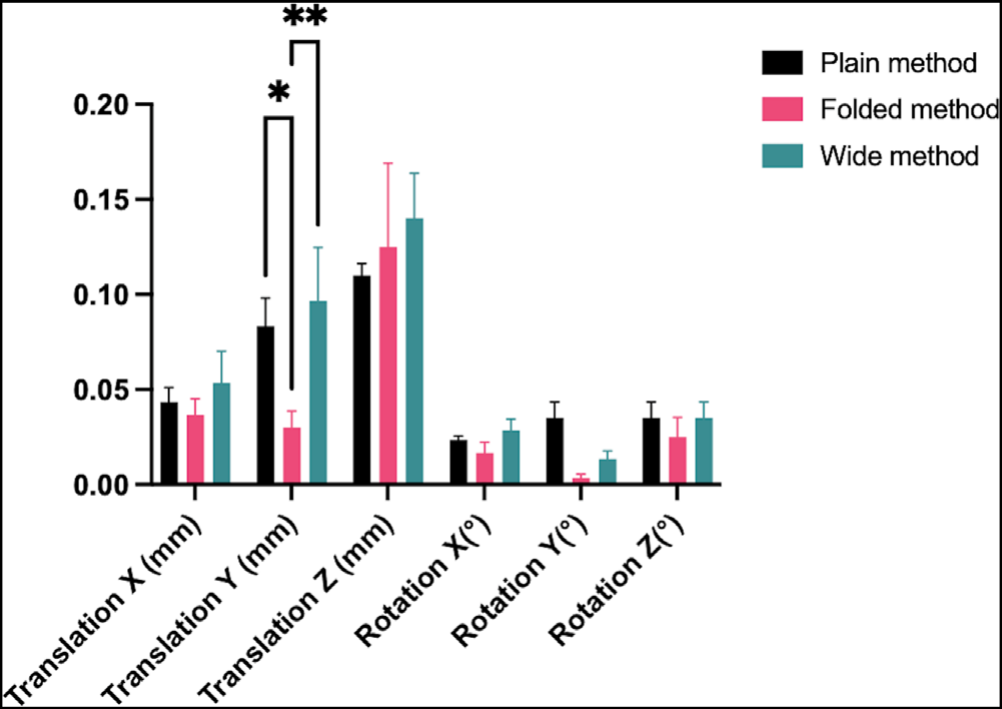
Inter‐fraction motions of the phantom during the initial application. *p < 0.05, **p < 0.01

Inter‐fraction motions were evaluated via the co‐registration of planning CBCT images. In the Leksell coordinate system, the X, Y, and Z axes extend from the right to the left of a patient, the posterior to the anterior, and the vertex to the feet, respectively. In the initial application, the translation for the Folded method (0.03 ± 0.02 mm) was significantly lower than that for the Plain (0.08 ± 0.03 mm, *p < 0.05) and Wide methods (0.09 ± 0.06 mm, **p < 0.01) among all the translational and rotational motions for each mask fixation (Figure 5). The rotations along all axes were under 0.1°, and no significant difference was observed in the rotations for each mask fixation method. During reapplications, the mask was carefully removed after the initial application, and the phantom position appeared to be affected by the pulling of mask buttons. Although the data of inter‐fraction motion showed low values (less than 0.6 in both translation [mm] and rotation [°] motion), we excluded the data because the position of the phantom could be changed by mask reapplication. The intra‐fraction motion result obtained using HDMM values remained at 0.1 mm for each mask fixation method in both the initial application and reapplications.

### 3.4 Temperature and humidity

In the initial application, the temperature rapidly increased from 20°C to 30°C for each mask fixation method. Then, the temperature rapidly decreased to the initial temperature of ∼20°C within 15 min. The humidity rapidly increased from 35% to 90%; however, it gradually decreased to the initial humidity within 1.5 h. No differences were observed between the temperature and humidity for each mask fixation method. Similarly, the temperature and humidity did not change during reapplications.

## 4 DISCUSSION

An evaluation of the characteristics of the three mask fixation methods revealed the following five interesting results:

1. Compared with the Plain and Wide methods, the largest force on the mandible was observed for the Folded method during the initial application and reapplications.
2. The force on the occipital bone increased during reapplications compared to that during the initial application for all mask fixation methods.
3. There were no statistically significant differences in the forces on each area when masks were reapplied.
4. The Folded method exhibited the least inter‐fraction motion in the initial application.
5. More than 1.5 h were required to completely stabilize the force and humidity during the initial application.

The coordinates of receiving the force were determined based on the Leksell system.^6^ During the initial application, the largest force acted on the occipital bone in the Plain and Wide methods, pushing down the patient's head along the Y‐axis. In the Folded method, the mandible was subjected to the largest force, while the occipital bone was subjected to a strong force (>4 N). The results of the Folded method implied that strong forces were applied to the X‐ and Y‐axes. The force on the occipital bone was stronger when the mask was reapplied in all mask fixation methods because the masks shrank. In addition, no significant force differences were observed in all mask fixation methods between the 1st and 2nd reapplications. If the patient felt discomfort during the 2nd reapplication, there could be other reasons (head positioning, general condition, facial swelling, etc.) other than the mask itself. Inata et al. showed that patient motion was reduced when the force on the occipital area was larger.^15^ As shown in Figure 3, the forces on the occipital bone at 1.5 h after the initial application were approximately 4 N in Plain and Folded methods. The force reached 80% of the maximum value in about 30 min in the Folded method, while it took about 1 h in the Plain and Wide methods. Furthermore, the Folded method showed a stronger force on the mandible area. Therefore, less patient motion can be expected with the Folded method than with the Plain and Wide methods, especially when the irradiation time is less than 1 h. When masks were reapplied, forces on the occipital bone were approximately 6 N in all fixation methods. However, we could expect suppression of patient movement in the Folded method because the force on the mandible was stronger with statistically significant differences. Patients could complain of more pressure on their occipital bone. As a remedy, in actual practice, a thin silicone pad can be placed on the head cushion if patients feel continuous discomfort on the occipital bone.^16^ All mask fixation methods showed low inter‐ and intra‐fraction motions. The phantom motion caused by the mask shrinkage or head cushion hardening was insignificant for all mask fixation methods. Based on our findings, all methods seem acceptable for frameless radiosurgery with the LGK Icon^TM^; however, the Folded method is found to exhibit the least inter‐fraction motion of translation along the Y‐axis. In addition, the pitching motion can be suppressed further in the Folded method compared with that in the other methods.

The technical data sheet for the Nanor mask (1.6 mm thickness) provides the following thermoforming conditions: the optimum activation temperature in a water bath (65°C), activation time in a water bath (3–4 min), hardening and working times (2 min), and time to completion (∼10 min). Ideally, the Nanor mask improves patient comfort by limiting patient movement through the reduction of contractions. The Nanor requires more than 1.5 h to completely stabilize during the execution of the three mask fixation methods. Therefore, it may be recommended that the patient wear a mask for 1.5 h before the first fraction of the treatment. However, for clinical application, it will be necessary to verify whether it is possible for the patient to tolerate the discomfort of wearing a mask beforehand and what are the benefits.

An important aspect of neurosurgery is precision planning based on individual characteristics and conditions.^17^, ^18^ GKRS is the most widely adopted method for treating brain tumors (malignant or benign) and vascular and functional diseases.^19^ GKRS requires precision planning for the fixation method. For each fraction, the patient undergoes a CBCT and the GammaPlan treatment planning system calculates the necessary shifts based on the co‐registration of the planning CBCT and pretreatment CBCT. While it is important for each reapplication of the mask to result in very similar patient positioning, the Icon's CBCT and registration process ensures that the patient setup is highly similar for each fraction. This can make up for any slight misalignment on a given day. Carminucci et al. analyzed the precision of frame and mask fixation methods using setup errors (inter‐fraction motion);^5^ they discovered that the motion variations in mask fixation were larger than those in frame fixation.^5^ The inter‐fraction motions for all mask fixation methods obtained in our study were similar to their frame fixation data. Although we analyzed the inter‐fraction motion through a phantom experiment, the inter‐fraction motion of the mask itself was extremely small in all mask fixation methods. In previous studies, mask fixation did not result in poor local control, systemic progression‐free survival, overall survival, or increased rates of radionecrosis for GKRS.^3^, ^7^, ^20^ Fixation using a mask or frame is determined on a case‐by‐case basis. In mask fixation, it is usually important to monitor the patient's intra‐fraction motion during irradiation.

In the Republic of Korea, there has been an increase in the demographic projections for the older population, resulting in higher cancer incidence and mortality.^21^ Accordingly, there is a surge in the number of cases requiring GKRS for brain metastases accompanied by eloquent areas or large lesions. Fractionated GKRS has become an effective treatment outcome owing to the introduction of the LGK Icon^TM^; this method results in minimal toxicity for large brain metastases compared to single‐session GKRS.^4^, ^22^ Mask fixation is commonly used owing to the increase in the number of patients undergoing fractionated GKRS. The Folded method is recommended if an unacceptable range of movement is expected due to the patient's condition, or if the patient feels uncomfortable with frame fixation for the treatment of a small lesion in an eloquent area. The Wide method is recommended for patient comfort, especially when the patient complains of a sense of closure and stuffiness with other methods. For all fixation methods, it would be better to notify the patient regarding the stronger force acting on the occipital bone during reapplications.

### 4.1 Limitations

This study has few limitations as listed below:

1. The phantom does not fully represent human skin; therefore, in future studies, we plan to create a 3D‐printed patient model that represents human skin.
2. We did not include the case of mask reapplication after reheating, and we plan to investigate the characteristics of reheated mask fixation in a future study.
3. We explicitly analyzed the characteristics of the Nanor mask. Additionally, we used a phantom; however, the inter‐ and intra‐fraction motion of patients is expected to be greater than that of the phantom.

## 5 CONCLUSION

The three mask fixation methods were found to provide adequate motion control. Based on our results, we recommend the Folded method for patients who require tight fixation and the Wide method for patients who experience suffocation. If fractionated GKRS is planned, the patients should be notified regarding the possibility of discomfort in the occipital bone during reapplications. Future work is planned to investigate the motion of patients according to the mask fixation methods.

## AUTHOR CONTRIBUTIONS

Hyeong Cheol Moon, Hyun‐Tai Chung, and Yun‐Sik Dho contributed to the design and the writing of the manuscript. Hyeong Cheol Moon, Hyun‐Tai Chung, and Byung Jun Min contributed to the data acquisition and analysis. Hyun‐Tai Chung and Yun‐Sik Dho checked the validity of the analysis and supervised the manuscript. All co‐authors approved the final manuscript.

## CONFLICT OF INTEREST

The authors report no conflicts of interest in this work.

## Data Availability

The data supporting the findings of this study are available from the corresponding author.
